# Ethanol Extracts from the Aerial Parts of *Inula japonica* and *Potentilla chinensis* Alleviate Airway Inflammation in Mice That Inhaled Particulate Matter 10 and Diesel Particulate Matter

**DOI:** 10.3390/nu15214599

**Published:** 2023-10-29

**Authors:** Seang-Hwan Jung, Kyung-Sook Chung, Chang-Seon Na, Hye-Shin Ahn, Yu-Kyong Shin, Kyung-Tae Lee

**Affiliations:** 1 Department of Biomedical and Pharmaceutical Sciences, College of Pharmacy, Kyung Hee University, Seoul 02247, Republic of Korea; i@khu.ac.kr; 2 Department of Pharmaceutical Biochemistry, College of Pharmacy, Kyung Hee University, Seoul 02247, Republic of Korea; adella76@khu.ac.kr; 3 Department of New Material Development, COSMAXBIO, Seongnam 13486, Republic of Korea; csna@cosmax.com (C.-S.N.); hsahn@cosmax.com (H.-S.A.)

**Keywords:** *Inula japonica*, *Potentilla chinensis*, airway inflammation, cytokines, pro-inflammatory proteins, particulate matter 10 (PM10), diesel particulate matter (DPM), PM10D

## Abstract

Air pollution causes various airway diseases. However, many commonly used treatments can have high risks of side effects or are costly. To examine the anti-inflammatory properties of *Inula japonica* Thunb. and *Potentilla chinensis* Ser., a mouse model was generated via inhalation of both particulate matter 10 and diesel particulate matter, and 30% ethanol extracts of either *I. japonica* (IJ) or *P. chinensis* (PC) and a mixture of both ethanol extracts (IP) were orally administered to BALB/c mice for 12 days. IJ, PC, and IP inhibited immune cell numbers and their regulation in both the bronchoalveolar lavage fluid (BALF) and lungs. These agents suppressed the levels of interleukin (IL)-1α, IL-17, tumor necrosis factor (TNF)-α, C-X-C motif chemokine ligand (CXCL)-1, and CXCL-2 in BALF, and also inhibited F4/80 and IL-1 receptor-associated kinase (IRAK)-1 in lungs. They reduced the gene expression of *TNF-α*, *CXCL-1*, *inducible NOS*, *COX-2*, *Mucin 5AC*, and *transient receptor potential cation channel subfamily V member 1* in lungs. These extracts also reduced histopathological changes and inflammatory progression, manifested as decreased cell infiltration, collagen deposition, and respiratory epithelial cell thickness. *I. japonica* and *P. chinensis* show potential for development as pharmaceuticals that suppress inflammatory progression and alleviate airway inflammation diseases caused by air pollutants.

## 1. Introduction

Air pollution has become a very serious risk factor and a major environmental threat to human health. Approximately 6.5 million people were dead globally in 2019 because of air contamination, making it the leading cause of mortality among all types of pollution, where approximately 9 million people were dead due to all pollution problems [1]. Mortality related to air pollution is increasing, and approximately nine of ten individuals living in the urban areas of around 1600 cities in 91 countries are affected by air pollution [1,2]. 

Air pollutants in urban areas consist of particulate matter (PM), carbon monoxide, nitrogen oxides, sulfur dioxide, and lead, which can cause serious illness and should be extensively regulated to protect the public [2]. Air pollutants can affect the human brain, eyes, nose, lungs, heart, liver, skin, gastrointestinal organs, urogenital organs, bones, immunological organs, and even mood; they are highly linked to various human diseases such as allergy, asthma, bronchitis, chronic obstructive pulmonary disease, lung cancer, systemic inflammation, atherosclerosis, cognitive dysfunction, impaired motor control, abortion, premature birth, poor sperm quality, social stress, sleep disorders, and emotional problems [2,3]. 

Coarse particulate matter (PM10) and fine particulate matter (PM2.5), with aerodynamic diameters of 2.5–10 and 0.1–2.5 µm, respectively, are very serious concerns along with diesel particulate matter (DPM) released from diesel-fueled vehicles. These particles are not filtered by the human upper airway but rather pass the upper and lower airways and enter the blood circulation to cause harmful effects throughout the body and cause disease [3,4]. DPM containing PM2.5 and PM10 can stimulate human inflammatory responses by releasing various pro-inflammatory cytokines and proteins such as interleukin (IL)-1β, IL-1 receptor-associated kinase 1 (IRAK1), tumor necrosis factor (TNF)-α, C-X-C motif chemokine ligand (CXCL)-1, CXCL-2, cyclooxygenase (COX)-2, or transient receptor potential cation channel subfamily V member 1 (TRPV1); increasing nitric oxide synthase (NOS) to generate oxidative stress; and inducing immune cell differentiation and activation [4,5]. 

Extracts from plants *Inula japonica* Thunb. and *Potentilla chinensis* Ser. were previously reported to have remedial effects by attenuating specific diseases such as allergy, asthma, hepatitis, hemorrhagic cystitis, and obesity [6,7,8,9,10]. However, whether these extracts can alleviate airway inflammation provoked by pollutants from the ambient air and their synergic effects remain unclear. Moreover, corticosteroids and long-acting β-agonists, which are commonly used to treat airway inflammation, can increase the risk of serious side effects [11,12]. IL-1 blockade has been considered for preventing lung cancer derived from airway inflammation, but this treatment is costly and its effectiveness is unclear [13]. Therefore, an affordable and effective therapy is needed. Therefore, in this study, *I. japonica* Thunb. and *P. chinensis* Ser., both individually and in combination, were evaluated for their pharmaceutical potential to alleviate airway inflammation in a mouse model prepared via inhalation of both PM10 and DPM (PM10D). The results were compared to those obtained in mice treated with dexamethasone.

## 2. Materials and Methods

### 2.1. Preparation of Ethanol Extracts from I. japonica and P. chinensis

The aerial parts of *I. japonica* Thunb. and *P. chinensis* Ser. were collected in Sunchang-gun, Jeollabuk-do, Republic of Korea, and classified by the MiDNA Genome Research Institute (Gunsan-si, Jeollabuk-do, Republic of Korea). Each sample was individually labeled as COS2008 and COS2009 and deposited at the herbarium of COSMAX BIO R&I Center (Seongnam-si, Gyeonggi-do, Republic of Korea). The aerial parts of the plants were washed with water and dried completely. They were extracted with 30% EtOH (30-fold *w*/*v*) at 80 °C for 5 h and concentrated up to 35 Brix under reduced pressure using a rotary evaporator. The extract residues were sterilized for 1 h to obtain 30% EtOH extracts of *I. japonica* Thunb. (IJ) and 30% EtOH extracts of *P. chinensis* Ser. (PC). IJ and PC were combined at a 1:1 ratio to prepare mixtures of two extracts. The mixture (IP) of two extracts was processed through a spray dryer to obtain a 30% EtOH extract mixture of IJ and PC at a 1:1 ratio as previously reported [10].

### 2.2. High-Performance Liquid Chromatography Analysis of Ethanol Extracts of IJ, PC, and IP

IJ (200 and 100 g), PC (200 and 100 g), and IP (200 and 100 g) were standardized as previously described [10]. Standard solutions of 2,3,4,5-tetracaffeoyl-D-glucaric acid and apigenin 7-*O*-*β*-D-glucuronide were prepared in a mixture of dimethyl sulfoxide and methanol (1:9 ratio, *v*/*v*) to a concentration of 2000 ppm. Each standard solution (500 μL) was aliquoted and gently mixed. Finally, the calibration standard mixture was serially diluted and adjusted to the following concentrations: 31.25, 62.5, 125, 250, 500, and 1000 ppm. The samples and calibration solutions were filtered through 0.2 μm Whatman PTFE membrane filters (Cat. WHA7582004, Merck, Kenilworth, NJ, USA) and analyzed using high-performance liquid chromatography (HPLC) on a Waters model 2695 HPLC pump with photodiode array detector (Waters model 2998) set to 365 nm (Waters, Milford, MA, USA) with a Gemini NX C18 110A (4.6 × 250 mm, 5 μm) (Cat. 00G-4435-E0, Phenominex, Torrance, CA, USA). The gradient mobile phases were 0.1% formic acid in acetonitrile (solvent A) and 0.1% formic acid in water (solvent B) with the following gradient elution as a percent of solvent A at a flow rate of 0.7 mL/min: 15% from 0 to 7 min; 15 to 20% from 7 to 27 min; 20% from 27 to 45 min; 20 to 25.4% from 45 to 52 min; 25.4 to 35% from 52 to 60 min; 35 to 43% from 60 to 73 min; and 43 to 100% from 73 to 74 min as previously reported [10].

### 2.3. Animals and Treatments

Male BALB/c mice, aged 6–8 weeks and weighing 19–22 g, were purchased from Orient Bio Co., Ltd. (Seongnam-si, Gyeonggi-do, Republic of Korea) and housed under standard laboratory conditions (light-dark cycle: 12 h, temperature of 22 ± 2 °C, humidity of 50 ± 10%) for 1 week. Water and food were provided *ad libitum*. The study protocol was approved by the Committee for Animal Welfare at Daejeon University (DJUARB2022-011, Approval date: 20 May 2022) and was performed in accordance with the animal guidelines of Daejeon University. Mice were orally administered the plant extracts every other day for 12 days and intranasal administration of a fine dust complex (PM10 containing arsenic, cadmium, lead, and nickel (Cat. ERMCZ120, Sigma-Aldrich, St. Louis, MO, USA) and DPM (Cat. NIST2975, Sigma-Aldrich)) on days 4, 7, and 10. For this study, 3 mg/mL of PM10 and 0.6 mg/mL of DPM were respectively dissolved in 1% aluminum hydroxide gel adjuvant and 99% saline, and nine groups of mice were prepared (*n* = 8 per group): (1) non-treated control group (N); (2) group that inhaled pollutants consisting of PM10D (P); (3) group treated with 3 mg/kg dexamethasone (Cat. D2915, Sigma-Aldrich, St. Louis, MO, USA) after inhalation of PM10D (DEX); (4) group treated with 200 mg/kg of IJ after inhalation of PM10D (IJ200); (5) group treated with 100 mg/kg of IJ after inhalation of PM10D (IJ100); (6) group treated with 200 mg/kg of PC after inhalation of PM10D (PC200); (7) group treated with 100 mg/kg of PC after inhalation of PM10D (PC100); (8) group treated with 200 mg/kg of IP after inhalation PM10D (IP200); and (9) group treated with 100 mg/kg of IP after inhalation of PM10D (IP100). The conditions of the mice, including their physical appearance, behavior, sensitivity, liveliness, and respiratory conditions, were observed daily. On day 12, all mice were euthanized, by using a 30% volume per minute displacement rate of 100% CO_2_ in an induction chamber (Harvard Apparatus, Holliston, MA, USA) and their blood, bronchoalveolar lavage fluid (BALF), trachea tissues, and lung tissues were collected.

### 2.4. Collection of Lung Cells and BALF and Cytological Analysis

Lung tissues were incubated in phosphate-buffered saline (PBS) containing 1 mg/mL collagenase IV (Cat. C5138, Sigma-Aldrich) at 37 °C for 40 min. The cell suspension was filtered and centrifuged at 450× *g* for 20 min to collect the cell pellets. BALF obtained via tracheotomy and tracheal cannulation was centrifuged at 400× *g* for 5 min at 4 °C. The collected lung and BALF samples were suspended in PBS, and total cell numbers were determined via fluorescence-activated cell sorting (FACS) analysis. For further cytological analysis, cells from the BALF were centrifuged, collected on cytospin slides at 400× *g* for 4 min, and stained with a modified Diff-Quik stain as previously reported [5].

### 2.5. Cell Counting by Cytospin

The cells were collected and stained with a Diff-Quick Stain Kit (Baxter Healthcare Corp., Miami, FL, USA) before counting using a hemocytometer as previously described [5]. 

### 2.6. Enzyme-Linked Immunosorbent Assay

Using enzyme-linked immunosorbent assay (ELISA) kits, the concentrations of IL-1α (Cat. DLA50), IL-17 (Cat. M1700), TNF-α (Cat. MTA00B), CXCL-1 (Cat. MKC00B), and CXCL-2 (Cat. MM200) in the BALF were determined (R&D Systems, Minneapolis, MN, USA). 

### 2.7. Quantitative Reverse Transcription Polymerase Chain Reaction (qRT-PCR)

Total RNAs were isolated and reverse-transcribed into cDNA using a First-Strand cDNA Synthesis kit (Cat. 27926101, Cyvita, Marlborough, MA, USA) with the primer and probe sequences listed in Table 1. Quantification was performed using SYBR Green PCR Master Mix (Applied Biosystems, Foster City, CA, USA) and a 7500 Real-Time PCR system (Applied Biosystems).

### 2.8. Flow Cytometry

Cells from the lungs and BALF were incubated with anti-CD3 (145-2C11), anti-CD4 (RM4-5), anti-CD69 (H1.2F3), anti-CD8 (53-6.7), anti-CD62L (MEL-14), anti-CD44 (IM7), anti-CD21/35 (7G6), anti-B220 (RA3-6B2), anti-CD206 (Y17-505), and anti-CD11c (HL3, 557400) antibodies obtained from BD Biosciences (San Diego, CA, USA) for 30 min, washed with PBS, and fixed with 0.5% paraformaldehyde solution for 20 min, and cells were analyzed via two-color flow cytometry on a FACS Caliber using CellQuest software v3.3 (BD Biosciences, San Diego, CA, USA) as previously described [5].

### 2.9. Immunofluorescence Staining

Frozen lung tissues were cut into 20-µm sections with a Cryostat Microtome (CM 3050S, Leica Microsystems, Wetzlar, Germany). The tissues were fixed with 4% paraformaldehyde and 4% sucrose in PBS at 20–25 °C for 40 min, permeabilized with IGEPAL CA-630 (Cat. I8896, Sigma-Aldrich) in PBS, and blocked with 2.5% horse serum and 2.5% bovine serum albumin at 20–25 °C for 16 h. For double immunofluorescence staining, the sections were exposed to antibodies against IL-1α (#50794, Cell Signaling Technology Inc., Danvers, MA, USA), F4/80 (#70076, Cell Signaling Technology Inc.), and IRAK-1 (ab238, Abcam, Cambridge, UK), at 4 °C overnight. A fluorescein-conjugated secondary antibody was added for 2 h, and Hoechst staining was performed as previously described [5]. The sections were visualized using an Eclipse Ti-E inverted fluorescence microscope (Nikon Instruments, Tokyo, Japan). The mean fluorescence intensity was quantified using images obtained from three independent experiments with ImageJ software v1.5.3 (NIH, Bethesda, MD, USA).

### 2.10. Histopathological Analysis of Tracheal and Lung Tissues

The lung and tracheal tissues were fixed in formalin, embedded in paraffin, and cut into 5-μm sections. They were stained with hematoxylin and eosin (H&E) and Masson’s trichrome (MT) as previously described [5].

### 2.11. Statistical Analysis

The data are presented as mean ± standard error of the mean (SEM) and analyzed using the ANOVA test in Prism software v7.0 (GraphPad Inc., San Diego, CA, USA). Values of *p* < 0.05 or less were considered statistically significant. Significant differences are denoted as ^#^ *p* < 0.05, ^##^ *p* < 0.01, and ^###^ *p* < 0.001 compared to the non-treated group (N), and * *p* < 0.05, ** *p* < 0.01, and *** *p* < 0.001 compared to the group which inhaled PM10D (P).

## 3. Results

### 3.1. Quantification of 2,3,4,5-tetracaffeoyl-D-glucaric Acid and Apigenin 7-O-β-D-glucuronide in IJ, PC, and IP

**Figure 1:**
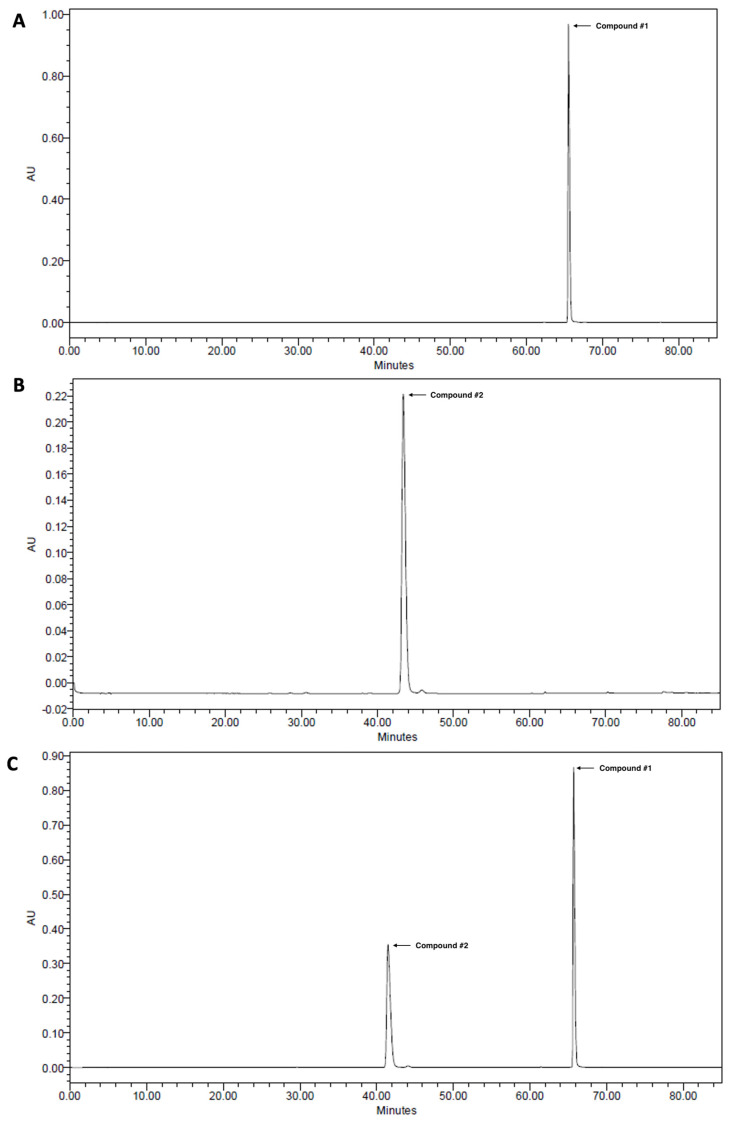

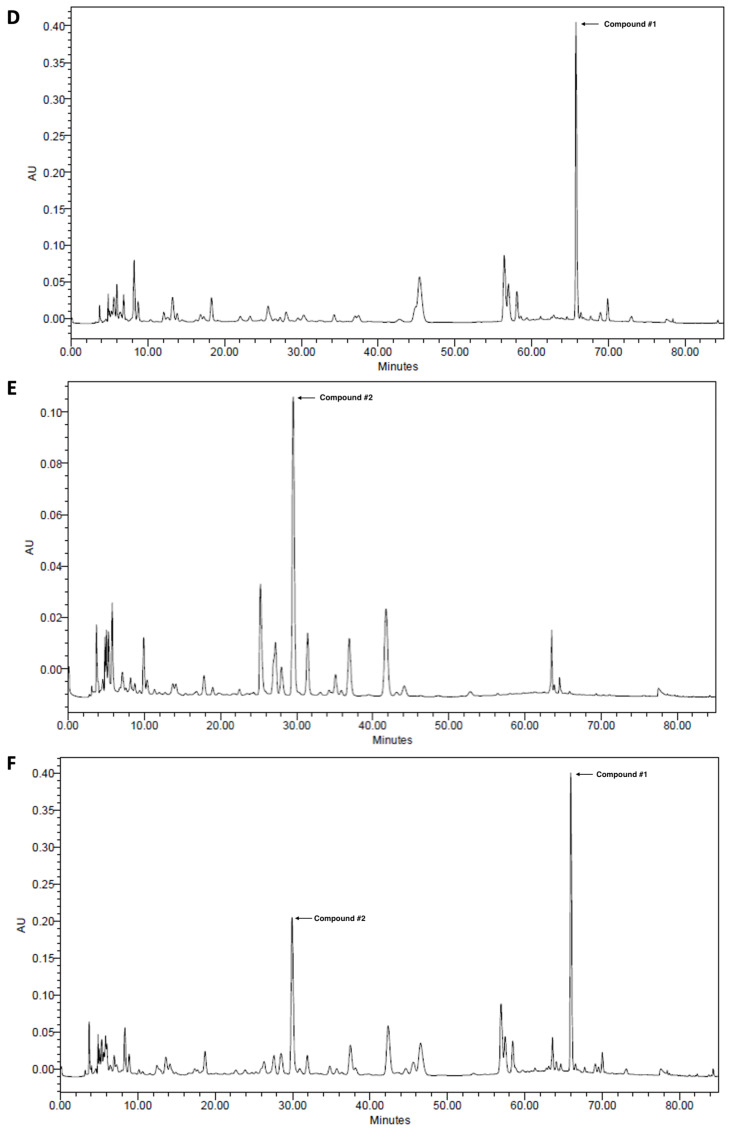
High-performance liquid chromatograms of (**A**) 2,3,4,5-tetracaffeoyl-D-glucaric acid (compound #**1**), (**B**) apigenin 7-*O*-*β*-D-glucuronide (compound #**2**), (**C**) mixture of 2,3,4,5-tetracaffeoyl-D-glucaric acid and apigenin 7-*O*-*β*-D-glucuronide, (**D**) 30% EtOH extracts of *I. japonica* Thunb. (IJ), (**E**) 30% EtOH extracts of *P. chinensis* Ser. (PC), and (**F**) 30% EtOH extracts of the mixture of IJ and PC at 1:1 ratio (IP).

2,3,4,5-Tetracaffeoyl-D-glucaric acid (compound #**1**) and apigenin 7-*O*-*β*-D-glucuronide (compound #**2**) were isolated from IJ and PC, respectively, and HPLC analyses were performed on each chemical and extract to determine whether IJ and PC contained compound #**1** and compound #**2** as previously reported [10]. Compounds #**1** and #**2** were detected at retention times (Rt) of 66.0 and 42.0 min, respectively in the standard (Figure 1A–C), and showed strong intensities for IJ, PC, and IP (Figure 1D–F). A peak for compound #**1** was detected in IJ and IP at the same retention time when compared to the standard, and its proportion in IJ was 20.0 mg/g (Figure 1D) and in IP was 10.0 mg/g (Figure 1F). A peak for compound #2 was also detected at the retention time of 30.0 min in both PC and IP when compared to the standard, and its proportions in PC and IP were 9.26 mg/g (Figure 1E) and 4.63 mg/g (Figure 1F), respectively. Both compounds were the major components in IJ, PC, and IP.

### 3.2. Effects of IJ, PC, and IP on Airway Immune Cell Numbers in the PM10D-Exposed Mice

**Figure 2:**
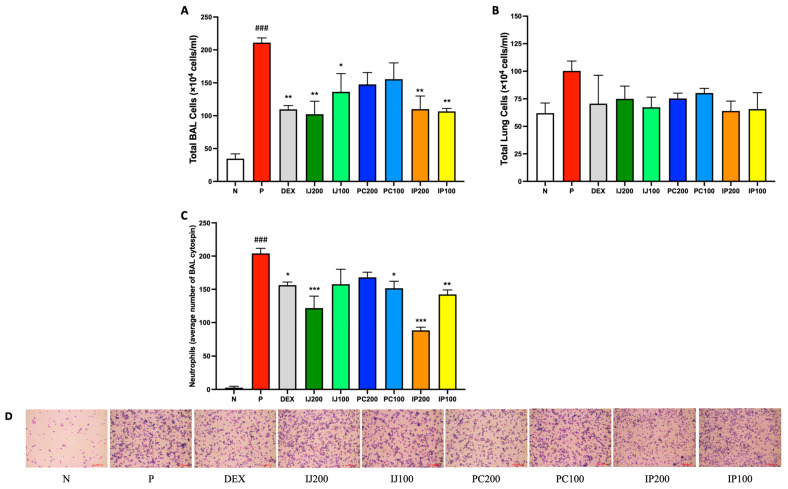
IJ, PC, IP, and dexamethasone reduced the number of airway immune cells in mice exposed to PM10D via inhalation. (**A**) Total BAL cell numbers, (**B**) total lung cell numbers, and (**C**) neutrophils in BALF centrifuged on cytospin slides were counted. (**D**) BALF centrifuged on cytospin slides was photographed at 200× magnification. The scale bar corresponds to a length of 100 μM. BAL: bronchoalveolar lavage; N: non-treated control group; P: group that inhaled pollutants consisting of PM10D; DEX: group treated with 3 mg/kg dexamethasone after inhalation of PM10D; IJ200: group treated with 200 mg/kg of IJ after inhalation of PM10D; IJ100: group treated with 100 mg/kg of IJ after inhalation of PM10D; PC200: group treated with 200 mg/kg of PC after inhalation of PM10D; PC100: group treated with 100 mg/kg of PC after inhalation of PM10D; IP200: group treated with 200 mg/kg of IP after inhalation of PM10D; IP100: group treated with 100 mg/kg of IP after inhalation of PM10D. Data are presented as the mean ± SEM, and significant differences are denoted as ^###^ *p* < 0.001 compared to the non-treated group (N); * *p* < 0.05, ** *p* < 0.01, and *** *p* < 0.001 compared to the group which inhaled PM10D (P).

The total cell numbers in the BALF and lungs of mice exposed to PM10D were potently reduced compared to those in the controls by treatment with IJ, PC, or IP (Figure 2A,B). Furthermore, IJ, PC, and IP reduced neutrophil infiltration in the BALF, and treatment with either IJ or IP at a higher concentration resulted in a more significant reduction in neutrophil infiltration compared to that observed using dexamethasone (Figure 2C,D). For both cell numbers and neutrophil infiltration, treatment with IJ200 and IP200 showed the greatest efficacy among treatments. 

### 3.3. Effects of IJ, PC, and IP on Immune Cell Regulation in the Lungs of PM10D-Exposed Mice

**Figure 3:**
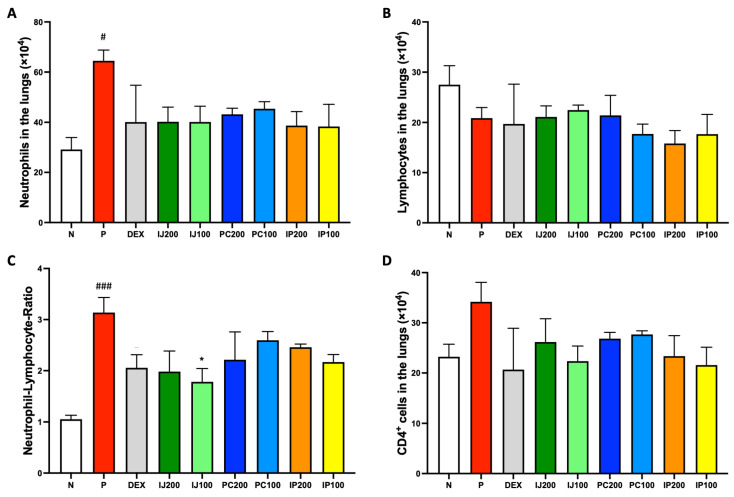

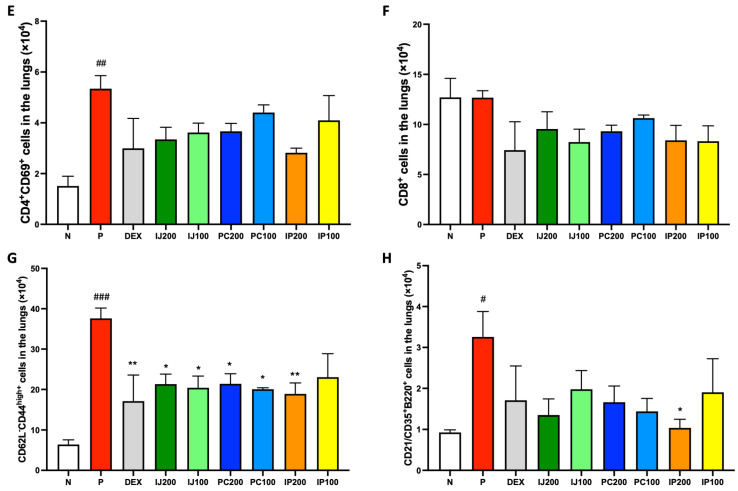
IJ, PC, IP, and dexamethasone suppressed the regulation of airway immune cells in the lungs of mice exposed to PM10D via inhalation. (**A**) Neutrophils, (**B**) lymphocytes, (**C**) neutrophil-lymphocyte ratio (NLR) value, (**D**) CD4^+^ helper T cells, (**E**) CD4^+^CD69^+^ T cells, (**F**) CD8^+^ cytotoxic T cells, (**G**) CD62L^-^CD44^high+^ memory T cells, and (**H**) CD21/CD35^+^B220^+^ marginal zone B cells were sorted and analyzed by FACS. N: Non-treated control group; P: group that inhaled pollutants consisting of PM10D; DEX: group treated with 3 mg/kg dexamethasone after inhalation of PM10D; IJ200: group treated with 200 mg/kg of IJ after inhalation of PM10D; IJ100: group treated with 100 mg/kg of IJ after inhalation of PM10D; PC200: group treated with 200 mg/kg of PC after inhalation of PM10D; PC100: group treated with 100 mg/kg of PC after inhalation of PM10D; IP200: group treated with 200 mg/kg of IP after inhalation of PM10D; IP100: group treated with 100 mg/kg of IP after inhalation of PM10D. Data are presented as the mean ± SEM, and significant differences are denoted as ^#^ *p* < 0.05, ^##^ *p* < 0.01, and ^###^ *p* < 0.001 compared to the non-treated group (N); * *p* < 0.05 and ** *p* < 0.01 compared to the group which inhaled PM10D (P).

IJ, PC, and IP influenced the activation and differentiation of various immune cells. According to FACS analysis of lung cells from mice exposed to PM10D, treatment with IJ, PC, and IP as well as dexamethasone tended to decrease the number of various inflammatory immune cells in the lungs. Neutrophils, which promptly respond during the acute inflammation phase, were significantly increased by PM10D but suppressed by treatment with dexamethasone, IJ, PC, and IP (Figure 3A). Lymphocytes, a type of white blood cells, were decreased by PM10D, dexamethasone, and each plant extract (Figure 3B). However, the neutrophil-to-lymphocyte ratio (NLR) value, which is the indicator of infection or inflammation, was increased by PM10D, whereas treatment with IJ, PC, or IP decreased the NLR value (Figure 3C), strongly indicating that IJ, PC, and IP exerted anti-inflammatory effects through the regulation of immune cells. Because the FACS results of total lymphocytes did not show distinct differences between PM10D and the plant extracts, subpopulations of lymphocytes were further sorted and analyzed using FACS (Figure 3D–H). IJ, PC, or IP suppressed various subsets of lymphocytes including CD4^+^ helper T cells (Figure 3D), CD4^+^CD69^+^ T cells (Figure 3E) for which treatment with IP200 showed a stronger effect than treatment with the other plant extracts and even dexamethasone, CD8^+^ cytotoxic T cells (Figure 3F), CD62L^-^CD44^high+^ memory T cells (Figure 3G), and CD21/CD35^+^B220^+^ marginal zone B cells (Figure 3H). 

### 3.4. Effects of IJ, PC, and IP on Immune Cell Regulation in BALF of PM10D-Exposed Mice

**Figure 4:**
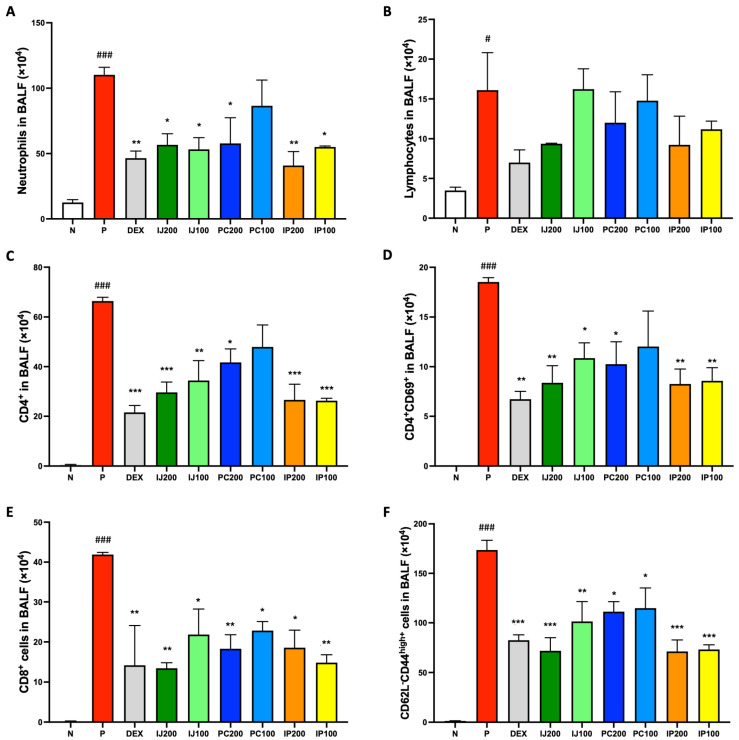
IJ, PC, IP, and dexamethasone suppressed activation and differentiation of airway immune cells in the BALF of mice exposed to PM10D via inhalation. (**A**) Neutrophils, (**B**) lymphocytes, (**C**) CD4^+^ helper T cells, (**D**) CD4^+^CD69^+^ T cells, (**E**) CD8^+^ cytotoxic T cells, and (**F**) CD62L^-^CD44^high+^ memory T cells were sorted and counted using FACS. N: Non-treated control group; P: group that inhaled pollutants consisting of PM10D; DEX: group treated with 3 mg/kg dexamethasone after inhalation of PM10D; IJ200: group treated with 200 mg/kg of IJ after inhalation of PM10D; IJ100: group treated with 100 mg/kg of IJ after inhalation of PM10D; PC200: group treated with 200 mg/kg of PC after inhalation of PM10D; PC100: group treated with 100 mg/kg of PC after inhalation of PM10D; IP200: group treated with 200 mg/kg of IP after inhalation of PM10D; IP100: group treated with 100 mg/kg of IP after inhalation of PM10D. Data are presented as the mean ± SEM, and significant differences are denoted as ^#^ *p* < 0.05 and ^###^ *p* < 0.001 compared to the non-treated group (N); * *p* < 0.05, ** *p* < 0.01, and *** *p* < 0.001 compared to the group which inhaled PM10D (P).

Because of the effects of ethanol extracts of *I. japonica* and *P. chinensis* on the regulation of immune cells in the lungs, their effects on immune cells in the BALF of mice were further investigated. Neutrophils, lymphocytes, and subsets of lymphocytes in the BALF were analyzed using FACS. Although exposure to PM10D significantly boosted the differentiation of various immune cells in the BALF of model mice, IJ, PC, and IP suppressed inflammatory effects induced by PM10D and induced anti-inflammatory effects (Figure 4). For example, neutrophils were significantly decreased by treatment with IJ, PC, and IP, with IP200 showing stronger effects than dexamethasone (Figure 4A). Lymphocytes were also decreased by treatment with the plant extracts, and the higher concentration of each extract showed better efficacy (Figure 4B). Furthermore, treatment with IJ, PC, and IP resulted in significant decreases in various subtypes of T cells in the BALF, such as CD4^+^ helper T cells, CD4^+^CD69^+^ T cells, CD8^+^ cytotoxic T cells, and CD62L^-^CD44^high+^ memory T cells (Figure 4C–F). Additionally, IJ and PC showed pronounced suppression of CD4^+^ helper T cells, CD8^+^ cytotoxic T cells, and CD206^+^CD11^c+^ macrophages in the mesenteric lymph nodes (MLN) of mice exposed to PM10D, whereas IP showed significant effects on CD206^+^CD11^c+^ macrophages only in the MLN of the mice (Figure S1).

### 3.5. Effects of IJ, PC, and IP on Pro-Inflammatory Cytokines and Chemokines in BALF of PM10D-Exposed Mice

**Figure 5:**
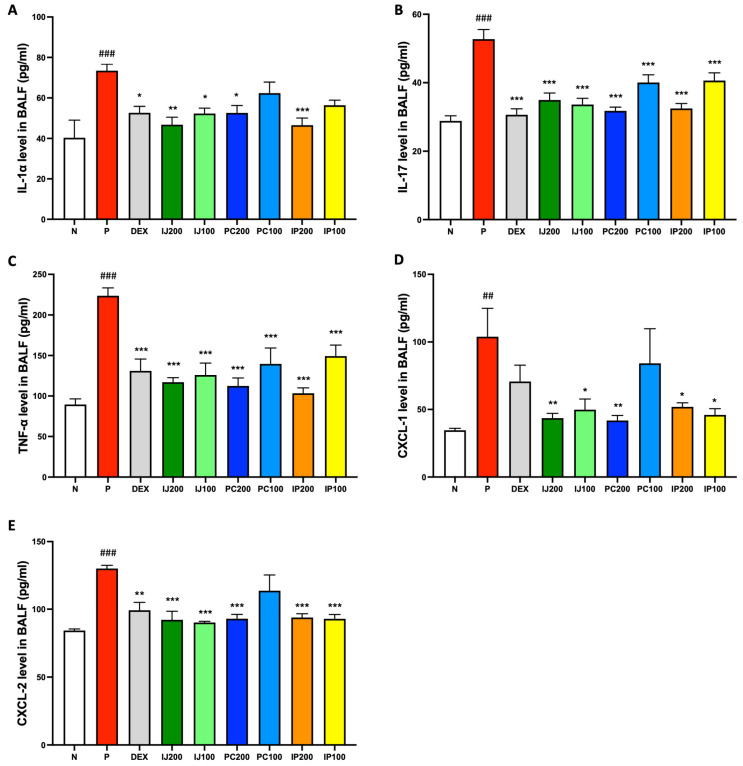
IJ, PC, IP, and dexamethasone inhibited pro-inflammatory proteins in the BALF of model mice exposed to PM10D. (**A**) Interleukin (IL)-1α, (**B**) IL-17, (**C**) TNF-α, (**D**) chemokine (C-X-C motif) ligand 1 (CXCL-1), and (**E**) CXCL-2 concentrations were measured using enzyme-linked immunosorbent assay. N: Non-treated control group; P: group that inhaled pollutants consisting of PM10D; DEX: group treated with 3 mg/kg dexamethasone after inhalation of PM10D; IJ200: group treated with 200 mg/kg of IJ after inhalation of PM10D; IJ100: group treated with 100 mg/kg of IJ after inhalation of PM10D; PC200: group treated with 200 mg/kg of PC after inhalation of PM10D; PC100: group treated with 100 mg/kg of PC after inhalation of PM10D; IP200: group treated with 200 mg/kg of IP after inhalation of PM10D; IP100: group treated with 100 mg/kg of IP after inhalation of PM10D. Data are presented as the mean ± SEM, and significant differences are denoted as ^##^ *p* < 0.01 and ^###^ *p* < 0.001 compared to the non-treated group (N); * *p* < 0.05, ** *p* < 0.01, and *** *p* < 0.001 compared to the group which inhaled PM10D (P).

Since IJ, PC, and IP modulated the PM10D-differentiated immune cells, we expected that IJ, PC, and IP also suppressed various pro-inflammatory cytokines and proteins in the PM10D-exposed mice. Based on the results of ELISA, inhalation exposure to PM10D significantly elevated the levels of several pro-inflammatory cytokines, including IL-1α, IL-17, and TNF-α, as well as chemokines such as CXCL-1 and CXCL-2 within the BALF of model mice, whereas IJ, PC, and IP suppressed PM10D-induced protein levels (Figure 5). Specifically, IL-1α was strongly inhibited by IJ200 and IP200 compared to the effects of other treatments (Figure 5A); IL-17 showed similar suppression levels when exposed to IJ200, IJ100, PC200, or IP200 (Figure 5B). Additionally, IJ200, IJ100, PC200, and IP200 demonstrated comparable effects to, or even greater effects than, dexamethasone against TNF-α (Figure 5C). Treatment with IJ, PC, and IP, but not PC100, significantly inhibited CXCL-1, surpassing the effects of dexamethasone (Figure 5D). The extracts showed comparable effects as dexamethasone against CXCL-2 (Figure 5E). IJ200 and IP200 most effectively suppressed the levels of pro-inflammatory cytokines and chemokines in the BALF of PM10D-exposed mice.

### 3.6. Effects of IJ, PC, and IP on Gene Expression of Pro-Inflammatory Mediators in the Lungs of PM10D-Exposed Mice

**Figure 6:**
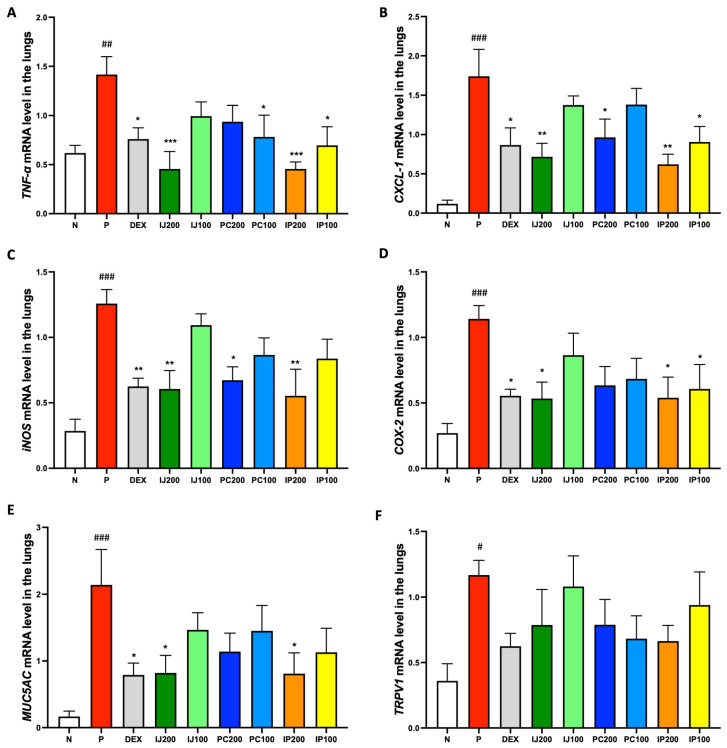
IJ, PC, IP, and dexamethasone inhibited gene expression of pro-inflammatory proteins in the lungs of mice exposed to PM10D via inhalation. (**A**) TNF-α, (**B**) CXCL-1, (**C**) inducible nitric oxide synthase (iNOS), (**D**) cyclooxygenase (COX)-2, (**E**) Mucin 5AC (MUC5AC), and (**F**) transient receptor potential cation channel subfamily V member 1 (TRPV1) mRNA were measured using qRT-PCR. N: Non-treated control group; P: group that inhaled pollutants consisting of PM10D; DEX: group treated with 3 mg/kg dexamethasone after inhalation of PM10D; IJ200: group treated with 200 mg/kg of IJ after inhalation of PM10D; IJ100: group treated with 100 mg/kg of IJ after inhalation of PM10D; PC200: group treated with 200 mg/kg of PC after inhalation of PM10D; PC100: group treated with 100 mg/kg of PC after inhalation of PM10D; IP200: group treated with 200 mg/kg of IP after inhalation of PM10D; IP100: group treated with 100 mg/kg of IP after inhalation of PM10D. Data are presented as the mean ± SEM, and significant differences are denoted as ^#^ *p* < 0.05, ^##^ *p* < 0.01 and ^###^ *p* < 0.001 compared to the non-treated group (N); * *p* < 0.05, ** *p* < 0.01, and *** *p* < 0.001 compared to the group which inhaled PM10D (P).

qRT-PCR analysis of mRNA levels confirmed that the extracts inhibited the transcription of pro-inflammatory genes. The mRNA levels of TNF-α, CXCL-1, inducible NOS (iNOS), COX-2, Mucin 5AC (MUC5AC), and TRPV1 in the lungs were measured (Figure 6). Similar to the results of protein level analyses, the qRT-PCR data showed that exposure to PM10D significantly upregulated the mRNA levels of various pro-inflammatory mediators in the mouse model, whereas IJ, PC, and IP suppressed PM10D-induced mRNA levels (Figure 6). All plant extracts suppressed TNF-α mRNA expression, with IJ 200 and IP200 showing stronger efficacy than dexamethasone (Figure 6A). The plant extracts inhibited transcription of CXCL-1; particularly, higher concentrations of samples showed greater efficacy, with IJ200 and IP200 showing significantly stronger effects than dexamethasone (Figure 6B). The plant extracts also suppressed the transcription of iNOS; higher concentrations showed greater efficacy, with IJ200, PC200, and IP200 exhibiting effects similar to those of dexamethasone (Figure 6C). Transcription of COX-2 and MUC5AC was inhibited by IJ, PC, and IP, with higher concentrations showing greater efficacy; particularly, IJ200 and IP200 showed equivalent effects to those of dexamethasone (Figure 6D–E). Finally, the plant extracts repressed the mRNA expression of TRPV-1, with IP200 showing a strong effect comparable to that of dexamethasone (Figure 6F). 

### 3.7. Effects of IJ, PC, and IP on IL-1α, F4/80, and IRAK1 in the Lungs of PM10D-Exposed Mice

**Figure 7:**
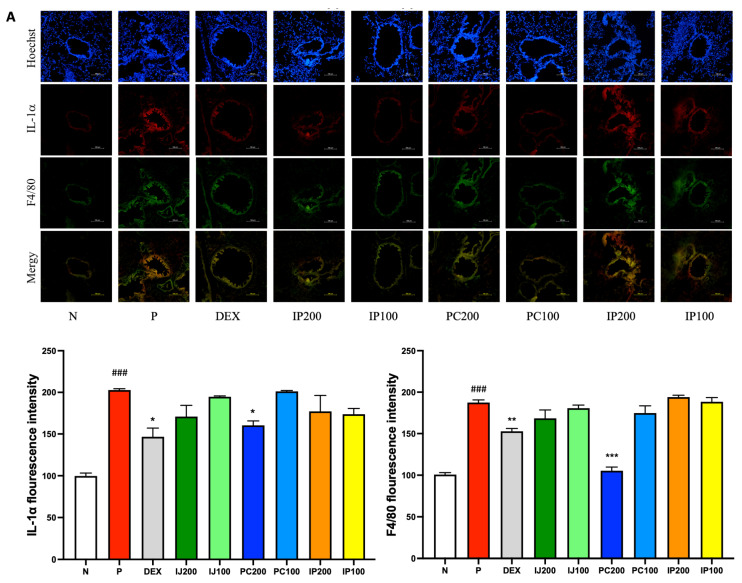

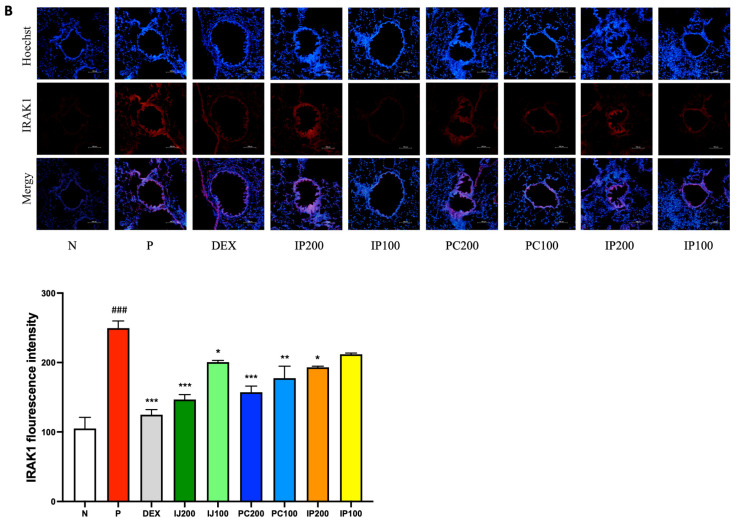
IJ, PC, IP, and dexamethasone suppressed the expression of IL-1α, F4/80, and interleukin-1 receptor-associated kinase 1 (IRAK1) in the lungs of mice exposed to PM10D. (**A**) IL-1α or F4/80 and (**B**) expression of IRAK1 were stained and visualized. N: Non-treated control group; P: group that inhaled pollutants consisting of PM10D; DEX: group treated with 3 mg/kg dexamethasone after inhalation of PM10D; IJ200: group treated with 200 mg/kg of IJ after inhalation of PM10D; IJ100: group treated with 100 mg/kg of IJ after inhalation of PM10D; PC200: group treated with 200 mg/kg of PC after inhalation of PM10D; PC100: group treated with 100 mg/kg of PC after inhalation of PM10D; IP200: group treated with 200 mg/kg of IP after inhalation of PM10D; IP100: group treated with 100 mg/kg of IP after inhalation of PM10D. The scale bar corresponds to a length of 100 μm. Data are presented as the mean ± SEM, and significant differences are denoted as ^###^ *p* < 0.001 compared to the non-treated group (N); * *p* < 0.05, ** *p* < 0.01, and *** *p* < 0.001 compared to the group which inhaled PM10D (P).

IL-1α, F4/80, and IRAK1 are involved in regulating inflammation. The immunohistofluorescence data showed that exposure to PM10D via inhalation significantly increased the levels of IL-1α, F4/80, and IRAK; the ethanol extracts of *I. japonica* and *P. chinensis* decreased the levels of these proteins (Figure 7). IL-1α and F4/80 were co-localized and similarly affected by treatment with IJ, PC, and IP; specifically, PC200 reduced F4/80 levels to a greater extent than that observed using dexamethasone (Figure 7A). IJ, PC, and IP suppressed the expression of IRAK1 protein, with IJ200 and PC200 showing the strongest effects (Figure 7B). IJ200 suppressed IRAK1 more effectively than IL-1α or F4/80 to induce anti-inflammatory effects in the lungs.

### 3.8. Effects of IJ, PC, and IP on Lung and Tracheal Tissue Damage in the PM10D-Exposed Mice

**Figure 8:**
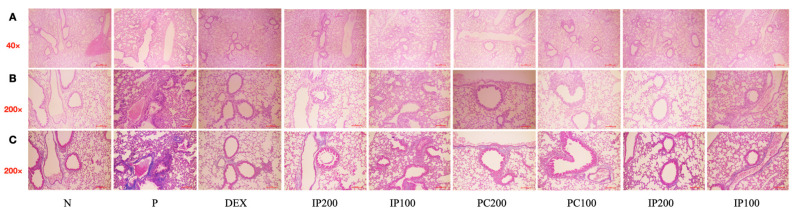
IJ, PC, IP, and dexamethasone significantly decreased histopathological changes and inflammation in the lungs of model mice exposed to PM10D. (**A**,**B**) Hematoxylin and eosin (H&E) staining at 40× and 200× magnification, (**C**) Masson’s trichrome (MT) staining shows inflammation around the bronchus and the mucus content. The scale bar corresponds to a length of 100 μm. N: Non-treated control group; P: group that inhaled pollutants consisting of PM10D; DEX: group treated with 3 mg/kg dexamethasone after inhalation of PM10D; IJ200: group treated with 200 mg/kg of IJ after inhalation of PM10D; IJ100: group treated with 100 mg/kg of IJ after inhalation of PM10D; PC200: group treated with 200 mg/kg of PC after inhalation of PM10D; PC100: group treated with 100 mg/kg of PC after inhalation of PM10D; IP200: group treated with 200 mg/kg of IP after inhalation of PM10D; IP100: group treated with 100 mg/kg of IP after inhalation of PM10D. The 100 µm scale bar corresponds to a length of 100 μm.

Histological analyses were conducted on lung and tracheal tissues using H&E staining and MT staining. The results showed that treatment with IJ, PC, and IP led to a significant decrease in histopathological changes and inflammation in the bronchial respiratory tissues (Figure 8). The treatments reduced cell infiltration, collagen deposition, and the thickness of respiratory epithelial cells, compared to the effects observed in mice that inhaled PM10D without treatment with IJ, PC, or IP. Histological analyses have confirmed that ethanol extracts from *I. japonica*, *P. chinensis*, and a combination of both extracts effectively suppressed histopathological changes, inflammatory progression, and lung damage in mice exposed to PM10D. 

## 4. Discussion

In the present study, the airway anti-inflammatory effects of IJ, PC, and IP were investigated to determine their pharmaceutical potential against the mouse model, which was generated through exposure to PM10D via inhalation. Currently, no papers or reports have been published investigating the collective anti-inflammatory effects of IJ, PC, or IP in the airway system of a mouse model exposed to air pollution. The other studies primarily focused on the individual effects of either IJ or PC against specific diseases such as allergy, asthma, hepatitis, hemorrhagic cystitis, or obesity without exploring their potential anti-inflammatory effects against inflammation caused by air pollution [6,7,8,9,10]. This study demonstrated, for the first time, that IJ and PC, both individually and in combination, have the potential to be developed into novel pharmaceuticals for suppressing inflammatory progression and alleviating airway inflammation diseases provoked by air pollutants in the ambient air. The previous work only studied the pharmaceutical potential of the mixture of IJ and PC in regulating specific conditions such as obesity [10], but this study extended the previous study and further illustrated that both the individual treatments of IJ or PC and the mixture of IJ and PC displayed pharmaceutical potential when evaluated side by side.

Overall, we showed that PM10D-provoked inflammation was alleviated and suppressed by IJ, PC, and IP. Dexamethasone is a synthetic glucocorticoid that mimics the effects of natural corticosteroids, is well-known for its anti-inflammatory properties, and is widely used in healthcare practice [14]. As inflammation is a complex process involving pro-inflammatory molecules that can be triggered by various external factors [15], specific inflammatory biomarkers were selected and analyzed before and after treatment with IJ, PC, and IP along with treatment of dexamethasone. The anti-inflammatory effects of IJ, PC, and IP were comparable to, or even more effective than, dexamethasone in suppressing the cellular and molecular biomarkers. For example, IJ, PC, and IP alleviated PM10D-induced inflammation by suppressing the differentiation and regulation of various immune cells, including neutrophils, lymphocytes, CD4^+^ helper T cells, CD4^+^CD69^+^ T cells, CD8^+^ cytotoxic T cells, CD62L^-^CD44^high+^ memory T cells, CD21/CD35^+^B220^+^ marginal zone B cells, and CD206^+^CD11^c+^ macrophages. Furthermore, IJ, PC, and IP improved the NLR value, a key indicator of the severity of inflammation and inflammatory diseases [16].

Correlations between inflammation and immune cell regulation are illustrated by pro-inflammatory cytokines and mediators, as these proteins initiate and amplify inflammation through the regulation of immune cells. IL-1α is a pro-inflammatory cytokine that promotes immune cell activation and recruitment [17]. IL-17 activates pro-inflammatory signaling cascades by upregulating inflammatory mediators and recruiting immune cells [18]. TNF-α is an important mediator that triggers cytokine releases and immune cell responses [19]. CXCL-1 and CXCL-2 are chemokines that mediate neutrophil functions via activation of CXCR-1 and CXCR-2, coupled to G-protein and β-arrestin-mediated signaling cascades [20]. iNOS orchestrates inflammatory progress via the NF-κB pathway [21]. COX-2 metabolizes arachidonic acid to form prostaglandin products, which trigger inflammation [22]. Lung MUC5AC is a marker of airway diseases such as asthma [23]. TRPV1, a channel protein found in immune cells such as lymphocytes, macrophages, and neutrophils, is implicated in immune responses including T cell activation [24]. F4/80 is a macrophage marker that plays a critical role in the innate immune response [25]. IRAK1 is a pivotal component for the IL-1α and Toll-like receptor signaling pathways [26]. Our results showed that IJ, PC, and IP not only effectively suppressed activation of the pro-inflammatory proteins described above, but also inhibited the transcriptional levels. Exposure to PM10D significantly increased the levels of various pro-inflammatory cytokines and mediators. In contrast, IJ, PC, and IP effectively countered the inflammatory effects of PM10D by suppressing the levels of various pro-inflammatory proteins such as IL-1α, IL-17, TNF-α, CXCL-1, and CXCL-2 and inhibiting the gene expression of *CXCL-1*, *iNOS*, *COX-2*, *MUC5AC*, and *TRPV1*. IJ200 suppressed IRAK1 more effectively than IL-1α or F4/80, whereas PC200 more strongly suppressed F4/80 levels, suggesting a reduction in the murine macrophage population; both IJ200 and PC200 displayed pronounced effects on IRAK1. Furthermore, IJ, PC, and IP reduced cell infiltration, collagen deposition, and the thickness of respiratory epithelial cells, indicating that they prevented histopathological changes, suppressed inflammatory progression, and reduced lung damage provoked by PM10D.

Due to the side effects and high risks associated with anti-inflammatory drugs, natural products have gained widespread attention as potential treatments for inflammation [27]. In the preclinical studies, the plant extracts did not exhibit any toxicity in rats. To determine the lethal dose and assess the potential toxicity of IJ, PC, and IP after a single administration in six-week-old male and female Sprague-Dawley rats (Study Number: 2301-1-451-2417, Shriram Institute for Industrial Research Tripartite Guidelines), all animals were monitored for clinical signs during a 14-day period. No animals succumbed during this period, and no unusual general clinical features were detected (data not shown). The lethal dose of IJ, PC, and IP exceeded 15,000 mg/kg in both male and female rats in this study. Consequently, the body weights of the experimental mice were not further monitored in this experiment. Furthermore, to evaluate the mutagenic potential of IJ, PC, and IP, histidine regulation in Salmonella typhimurium (TA98, TA100, TA102, TA1535, and TA1537) was assessed in the presence or absence of metabolic activation (Study Number: 2301-1-451-2415, Shriram Institute for Industrial Research Guidelines). The mean number of revertant colonies was consistently less than twice that of the negative control group at all dose levels of IJ, PC, and IP, up to doses of 5000 µg/plate, in all strains, both in the presence and absence of metabolic activation. In this study, IJ, PC, and IP did not exhibit any indication of mutagenic potential.

The HPLC analysis identified 2,3,4,5-tetracaffeoyl-D-glucaric acid and apigenin 7-*O*-*β*-D-glucuronide as the major compounds in IJ, PC, and IP, both of which have been reported to possess anti-inflammatory effects [28,29]. It is assumed that these compounds play crucial roles in the anti-inflammatory effects of IJ, PC, and IP. However, a detailed analysis of the remaining components in IJ, PC, and IP should be conducted in the subsequent study. Direct comparisons between natural products and dexamethasone, a synthetic chemical, can be ambiguous due to the presence of various unidentified compounds in the plants. However, dexamethasone can lead to severe side effects when used over an extended period, highlighting the urgent need for the development of safer alternatives. In this study, IJ, PC, and IP demonstrated comparable or preferable effectiveness to 3 mg/kg of dexamethasone in this study. It has been reported that 3.3 mg/kg of dexamethasone is a sufficient concentration to protect mice from severe inflammation induced by *Listeria monocytogenes* [30]. The anti-inflammatory effects of IJ, PC, and IP were comparable to those of 3 mg/kg of dexamethasone in this study. Because the natural compounds having various components can exhibit pharmacological effects irrespective of dosage, to explore the concentration-dependent effects of IJ, PC, and IP, as well as to determine their optimal concentrations for inflammation treatment, further investigations are needed at various concentrations. These future studies will help to establish the therapeutic window of IJ, PC, and IP in comparison to different concentrations of dexamethasone. 

Evaluation of the anti-inflammatory effects of ethanol extracts from *I. japonica* and *P. chinensis,* as well as a mixture of two plant extracts, demonstrated that PM10D induced airway inflammation in a mouse model exposed to PM10D through inhalation, and the plant extracts effectively counteracted and suppressed PM10D-provoked inflammation. These plant extracts reduced cell numbers, decreased the NLR value, inhibited immune cells, suppressed pro-inflammatory cytokines and mediators, and prevented histopathological changes and lung damage. These results strongly indicate that the plant extracts have important pharmaceutical potential for suppressing inflammatory progression, treating airway inflammation, and reducing lung damage. Therefore, ethanol extracts of *I. japonica* and *P. chinensis*, as well as a mixture of two plant extracts, show potential as affordable and effective therapeutic agents for managing airway inflammation diseases provoked by pollutants in the ambient air.

## 5. Conclusions

The ethanol extracts of the plants *I. japonica* and *P. chinensis*, as well as a mixture of the two plant extracts, showed anti-inflammatory effects that counteracted and suppressed PM10D-provoked airway inflammation. The plant extracts reduced cell numbers, decreased the NLR value, inhibited immune cells, suppressed inflammatory cytokines and mediators, and prevented histopathological changes and lung damage. These results strongly suggest that these plant extracts have pharmaceutical potential for suppressing inflammatory progression, treating airway inflammation, and reducing lung damage. Furthermore, the anti-inflammatory effects of the plant extracts were comparable or even preferable to those of 3 mg/kg of dexamethasone, a commonly used strong anti-inflammatory medication. Therefore, ethanol extracts of *I. japonica* and *P. chinensis*, as well as a mixture of two plant extracts, can be used as a promising health supplement for managing airway inflammation diseases provoked by pollutants in the ambient air. 

## Figures and Tables

**Table 1:** Sequences of the primers and probe used.

Gene		Sequences
*IL-1α*	Forward	5′-CAGGGTGGGTGTGCCGTCTTTC-3′
	Reverse	5′-TGCTTCCAAACCTTTGACCTGGGC-3′
*IL-17*	Forward	5′- TCTCATCCAGCAAGAGATCC-3′
	Reverse	5′- AGTTTGGGACCCCTTTACAC-3′
*CXCL-1*	Forward	5′-TCTCAGCACCCACCCGCTCA-3′
	Reverse	5′-GCCCCGTAGACCCTGCTCGA-3′
*CXCL-2*	Forward	5′-TCTCAGCACCCACCCGCTCA-3′
	Reverse	5′-GCCCCGTAGACCCTGCTCGA-3′
*TNF-α*	Forward	5′-TTGACCTCAGCGCTGAGTTG-3′
	Reverse	5′-CCTGTAGCCCACGTCGTAGC-3′
*iNOS*	Forward	5′-GCAGCTGAATGGAAAGATCA-3′
	Reverse	5′-TCCAGGAGACGTACAACAAT-3′
*COX-2*	Forward	5′-TCTCAGCACCCACCCGCTCA-3′
	Reverse	5′-GCCCCGTAGACCCTGCTCGA-3′
*MUC5AC*	Forward	5′-AGAATATCTTTCAGGACCCCTGCT-3′
	Reverse	5′-ACACCAGTGCTGAGCATACTTTT-3′
*TRPV1*	Forward	5′-TTGGATTTTCCACAGCCGTAGT-3′
	Reverse	5′-CAGACAGGATCTCTCCAGTGAC-3′
*GAPDH*	Forward	5′-CAATGAATACGGCTACAGCAAC-3′
	Reverse	5′-AGGGAGATGCTCAGTGTTGG-3′

## Data Availability

Not applicable.

## Supplementary Materials

The following supporting information can be downloaded at: https://www.mdpi.com/article/10.3390/nu15214599/s1, Figure S1: IJ, PC, IP, and dexamethasone suppressed activation and differentiation of airway immune cells in the MLN of mice exposed to PM10D via inhalation..

## References

[1] Fuller R., Landrigan P.J., Balakrishnan K., Bathan G., Bose-O’Reilly S., Brauer M., Caravanos J., Chiles T., Cohen A., Corra L. (2022). Pollution and health: A progress update. Lancet Planet Health.

[2] Kurt O.K., Zhang J., Pinkerton K.E. (2016). Pulmonary health effects of air pollution. Curr. Opin. Pulm. Med..

[3] Schraufnagel D.E., Balmes J.R., Cowl C.T., De Matteis S., Jung S.H., Mortimer K., Perez-Padilla R., Rice M.B., Riojas-Rodriguez H., Sood A. (2019). Air Pollution and Noncommunicable Diseases: A Review by the Forum of International Respiratory Societies’ Environmental Committee, Part 2: Air Pollution and Organ Systems. Chest.

[4] Hantrakool S., Kumfu S., Chattipakorn S.C., Chattipakorn N. (2022). Effects of Particulate Matter on Inflammation and Thrombosis: Past Evidence for Future Prevention. Int. J. Environ. Res. Public Health.

[5] Lee Y.S., Yang W.K., Park Y.R., Park Y.C., Park I.J., Lee G.J., Kang H.S., Kim B.K., Kim S.H. (2022). Opuntia ficus-indica Alleviates Particulate Matter 10 Plus Diesel Exhaust Particles (PM10D)-Induced Airway Inflammation by Suppressing the Expression of Inflammatory Cytokines and Chemokines. Plants.

[6] Lee E., Kim S.G., Park N.Y., Park H.H., Jeong K.T., Choi J., Lee I.H., Lee H., Kim K.J., Lee E. (2016). KOTMIN13, a Korean herbal medicine alleviates allergic inflammation in vivo and in vitro. BMC Complement. Altern. Med..

[7] Park Y.N., Lee Y.J., Choi J.H., Jin M., Yang J.H., Li Y., Lee J., Li X., Kim K.J., Son J.K. (2011). Alleviation of OVA-induced airway inflammation by flowers of Inula japonica in a murine model of asthma. Biosci. Biotechnol. Biochem..

[8] Juszczak K., Adamowicz J., Zapała L., Kluz T., Adamczyk P., Wdowiak A., Bojar I., Misiek M., Grzybowska M.E., Stangel-Wójcikiewicz K. (2022). Potentilla chinensis aqueous extract attenuates cyclophosphamide-induced hemorrhagic cystitis in rat model. Sci. Rep..

[9] Chen S., Huang Y., Su H., Zhu W., Wei Y., Long Y., Shi Y., Wei J. (2022). The Integrated Analysis of Transcriptomics and Metabolomics Unveils the Therapeutical Effect of Asiatic Acid on Alcoholic Hepatitis in Rats. Inflammation.

[10] Lee S.Y., Chung K.S., Son S.R., Lee S.Y., Jang D.S., Lee J.K., Kim H.J., Na C.S., Lee S.H., Lee K.T. (2022). A Botanical Mixture Consisting of Inula japonica and Potentilla chinensis Relieves Obesity via the AMPK Signaling Pathway in 3T3-L1 Adipocytes and HFD-Fed Obese Mice. Nutrients.

[11] Heffler E., Madeira L.N.G., Ferrando M., Puggioni F., Racca F., Malvezzi L., Passalacqua G., Canonica G.W. (2018). Inhaled Corticosteroids Safety and Adverse Effects in Patients with Asthma. J. Allergy Clin. Immunol. Pract..

[12] Casale T.B., Foggs M.B., Balkissoon R.C. (2022). Optimizing asthma management: Role of long-acting muscarinic antagonists. J. Allergy Clin. Immunol..

[13] Gottschlich A., Endres S., Kobold S. (2018). Can we use interleukin-1β blockade for lung cancer treatment?. Transl. Lung Cancer Res..

[14] Noreen S., Maqbool I., Madni A. (2021). Dexamethasone: Therapeutic potential, risks, and future projection during COVID-19 pandemic. Eur. J. Pharmacol..

[15] Brightling C., Greening N. (2019). Airway inflammation in COPD: Progress to precision medicine. Eur. Respir. J..

[16] Buonacera A., Stancanelli B., Colaci M., Malatino L. (2022). Neutrophil to Lymphocyte Ratio: An Emerging Marker of the Relationships between the Immune System and Diseases. Int. J. Mol. Sci..

[17] Balázs A., Mall M.A. (2019). Mucus obstruction and inflammation in early cystic fibrosis lung disease: Emerging role of the IL-1 signaling pathway. Pediatr. Pulmonol..

[18] Ritzmann F., Lunding L.P., Bals R., Wegmann M., Beisswenger C. (2022). IL-17 Cytokines and Chronic Lung Diseases. Cells.

[19] Muth K.N., Rech J., Losch F.O., Hoerning A. (2023). Reversing the Inflammatory Process-25 Years of Tumor Necrosis Factor-α Inhibitors. J. Clin. Med..

[20] Rajarathnam K., Schnoor M., Richardson R.M., Rajagopal S. (2019). How do chemokines navigate neutrophils to the target site: Dissecting the structural mechanisms and signaling pathways. Cell Signal..

[21] Nakazawa H., Chang K., Shinozaki S., Yasukawa T., Ishimaru K., Yasuhara S., Yu Y.M., Martyn J.A., Tompkins R.G., Shimokado K. (2017). iNOS as a Driver of Inflammation and Apoptosis in Mouse Skeletal Muscle after Burn Injury: Possible Involvement of Sirt1 S-Nitrosylation-Mediated Acetylation of p65 NF-κB and p53. PLoS ONE.

[22] Jung H.Y., Yoo D.Y., Nam S.M., Kim J.W., Kim W., Kwon H.J., Lee K.Y., Choi J.H., Kim D.W., Yoon Y.S. (2019). Postnatal changes in constitutive cyclooxygenase-2 expression in the mice hippocampus and its function in synaptic plasticity. Mol. Med. Rep..

[23] Cho H.Y., Park S., Miller L., Lee H.C., Langenbach R., Kleeberger S.R. (2021). Role for Mucin-5AC in Upper and Lower Airway Pathogenesis in Mice. Toxicol. Pathol..

[24] Singer J.W., Fleischman A., Al-Fayoumi S., Mascarenhas J.O., Yu Q., Agarwal A. (2018). Inhibition of interleukin-1 receptor-associated kinase 1 (IRAK1) as a therapeutic strategy. Oncotarget.

[25] Cassado A.D.A. (2017). F4/80 as a Major Macrophage Marker: The Case of the Peritoneum and Spleen. Results Probl. Cell Differ..

[26] Bujak J.K., Kosmala D., Szopa I.M., Majchrzak K., Bednarczyk P. (2019). Inflammation, Cancer and Immunity-Implication of TRPV1 Channel. Front. Oncol..

[27] Kishore N., Kumar P., Shanker K., Verma A.K. (2019). Human disorders associated with inflammation and the evolving role of natural products to overcome. Eur. J. Med. Chem..

[28] Qi C., Wang E., Jin L., Yan M., Zhang X., Wang H., Ye W. (2017). Ent-kaurene diterpenoids and lignan from *Leontopodium leontopodioides* and their inhibitory activities against cyclooxygenases-1 and 2. Phytochem Lett..

[29] Hu W., Wang X., Wu L., Shen T., Ji L., Zhao X., Si C., Jiang Y., Wang G. (2016). Apigenin-7-O-β-D-glucuronide inhibits LPS-induced inflammation through the inactivation of AP-1 and MAPK signaling pathways in RAW 264.7 macrophages and protects mice against endotoxin shock. Food Funct..

[30] Nakane A., Okamoto M., Asano M., Kohanawa M., Satoh Y., Minagawa T. (1994). Protection by dexamethasone from a lethal infection with Listeria monocytogenes in mice. FEMS Immunol. Med. Microbiol..

