# Supplementary material for: Ethanol Extracts from the Aerial Parts of Inula japonica and Potentilla chinensis Alleviate Airway Inflammation in Mice That Inhaled Particulate Matter 10 and Diesel Particulate Matter
Source: Nutrients. 2023 Oct 29;15(21):4599. doi: 10.3390/nu15214599 (PMC10647664; doi:10.3390/nu15214599)
Supplement: Supplementary file 1 [file nutrients-15-04599-s001.zip › nutrients-2627765-supplementary.pdf]

nutrients

MDPI

Supplementary Materials

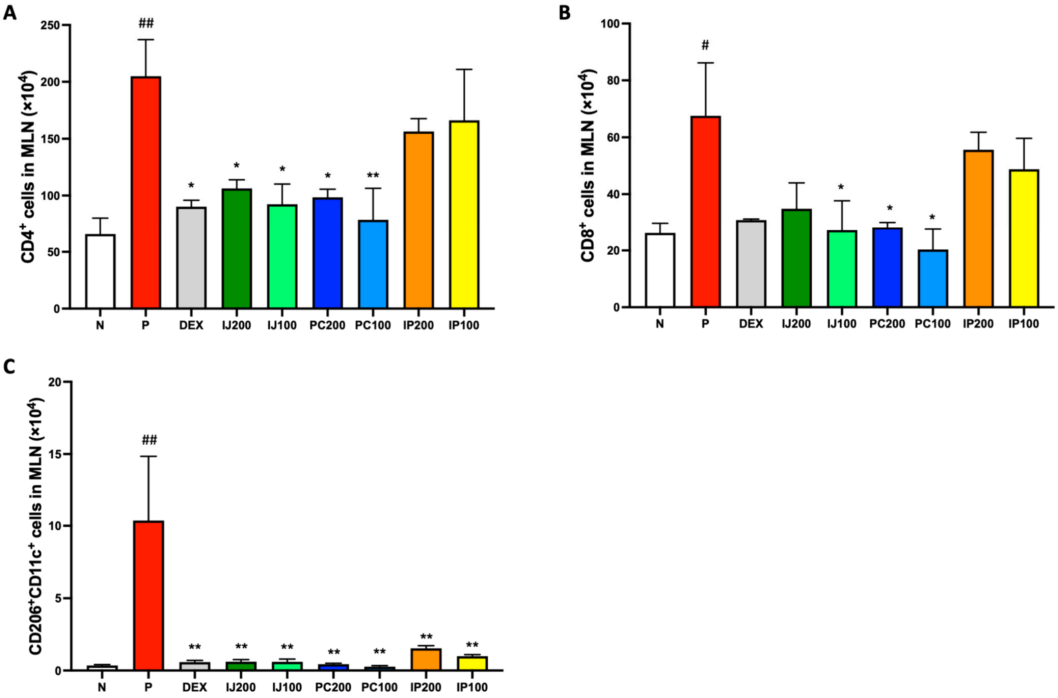

Figure S1. IJ, PC, IP, and dexamethasone suppressed activation and differentiation of airway immune cells in the MLN of mice exposed to PM10D via inhalation. **(A)** $CD4^+$ helper T cells, **(B)** $CD8^+$ cytotoxic T cells, and **(C)** $CD206^+CD11^{c+}$ macrophages were sorted and counted using fluorescence-activated cell sorting. N: Non-treated control group; P: group that inhaled pollutants consisting of PM10D; DEX: group treated with 3 mg/kg dexamethasone after inhalation of PM10D; IJ200: group treated with 200 mg/kg of IJ after inhalation of PM10D; IJ100: group treated with 100 mg/kg of IJ after inhalation of PM10D; PC200: group treated with 200 mg/kg of PC after inhalation of PM10D; PC100: group treated with 100 mg/kg of PC after inhalation of PM10D; IP200: group treated with 200 mg/kg of IP after inhalation of PM10D; IP100: group treated with 100 mg/kg of IP after inhalation of PM10D. Data are presented as the mean ± SEM, and significant differences are denoted as $^{\#}p < 0.05$ and $^{\#\#}p < 0.01$ compared to the non-treated group (N); $^{*}p < 0.05$ and $^{**}p < 0.01$ compared to the group which inhaled PM10D (P).
